# Supplementary material for: Development and Characterizations of Engineered Electrospun Bio-Based Polyurethane Containing Essential Oils
Source: Membranes (Basel). 2022 Feb 10;12(2):209. doi: 10.3390/membranes12020209 (PMC8876489; doi:10.3390/membranes12020209)
Supplement: Supplementary file 1 [file membranes-12-00209-s001.zip › membranes-1557204-supplementary.pdf]

Development and characterizations of engineered electrospun bio-based polyurethane containing essential oils

Nehir Arik[a], Nesrin Horzum[a,b]* and Yen Bach Truong[c]

[a] *Department of Biocomposite Engineering Graduate Program, Izmir Katip Celebi University, Graduate School of Natural and Applied Sciences, Izmir, Turkey*

[b] *Department of Engineering Sciences, Izmir Katip Celebi University, Faculty of Engineering and Architecture, Izmir, Turkey*

[c] *CSIRO Manufacturing, Bag 10, Clayton, Victoria, 3168, Australia*

*Corresponding author: E-mail: nesrin.horzum.polat@ikcu.edu.tr

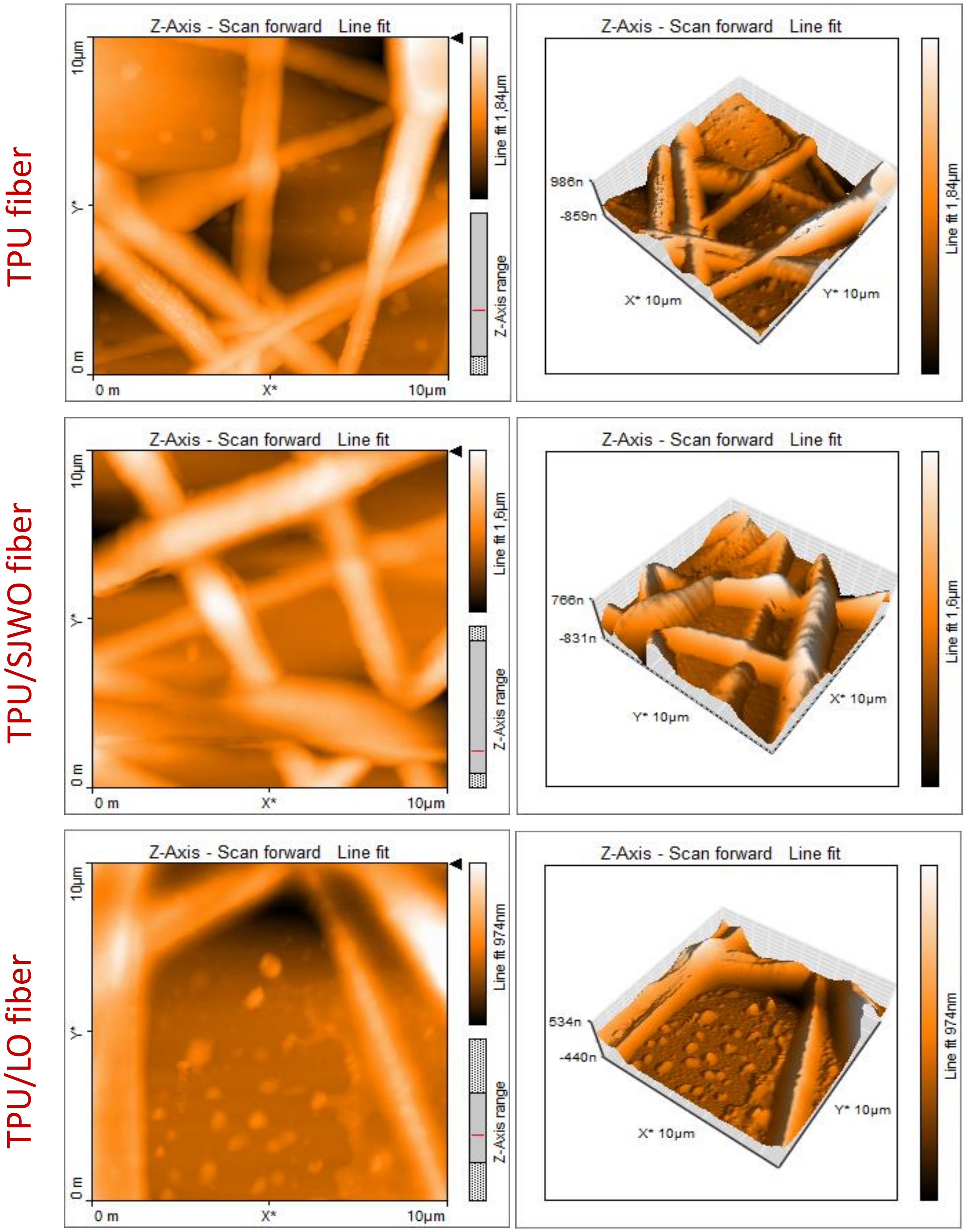

Figure S1. AFM images of 12.5 wt % biobased TPU, TPU/SJWO (20 wt %) and TPU/LO (20 wt %) fibers (Applied voltage: 10.0 kV, Flow rate: 1.00 mL h^{-1}, Distance: 17 cm)

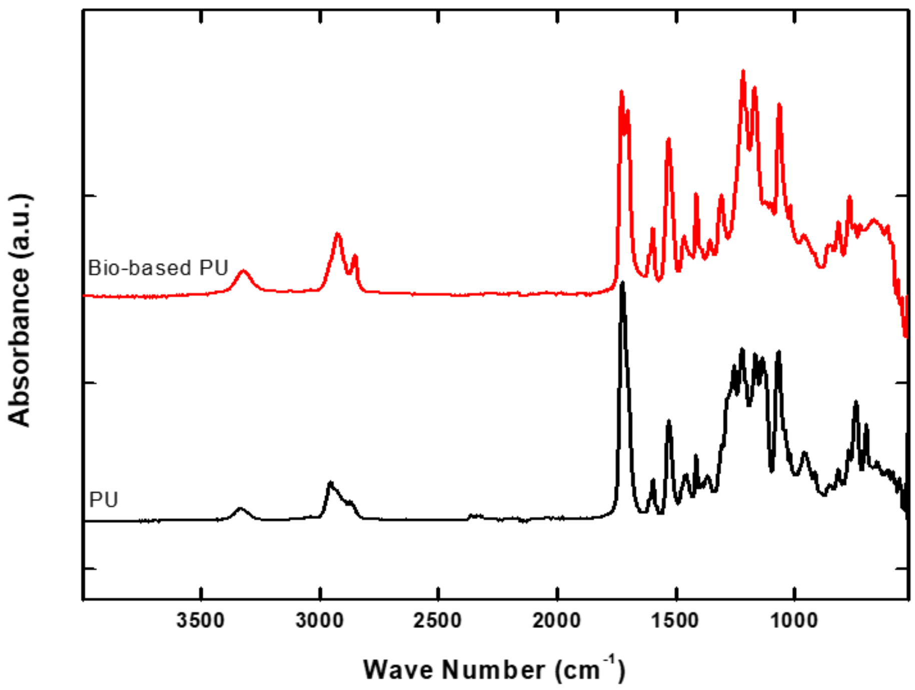

Figure S2. FTIR spectra of 12.5 wt % biobased TPU and commercial TPU nanofibers (Applied voltage: 12.5 kV, Flow rate: 1.00 mL h^{-1}, Distance: 17 cm)

Table S1: Viscosity (μ) of TPU/DMF solutions

TPU concentrations (wt %) in DMF	μ [mPa·s]
5	66.8
10	136
12.5	439
15	588

Table S2: Characteristics of surface roughness parameters (The fibers were obtained at applied voltage: 10.0 kV, flow rate: 1.00 mL h^{-1}, and distance: 17 cm, TPU concentration in the solutions was 12.5 wt %)

Fiber	Sa (3D roughness average, nm)	Sq (root mean square roughness, nm)	Contact angle (°)
**TPU**	289	355	91±2.0
**TPU/SJWO**	227	275	87±3.0
**TPU/LO**	121	160	77±4.0
